# CLIPPERS: Multiparametric and quantitative MRI features

**DOI:** 10.1016/j.radcr.2022.10.043

**Published:** 2022-11-17

**Authors:** Alexandra M. Korostyshevskaya, Julia A. Stankevich, Liubov M. Vasilkiv, Olga B. Bogomyakova, Denis S. Korobko, Alyona M. Gornostaeva, Andrey А. Tulupov

**Affiliations:** a The Institute International Tomography Center of the Russian Academy of Sciences, Institutskaya str., Bldg. 3а, Novosibirsk, 630090, Russian Federation; b Federal State Budgetary Scientific Institution «The Federal Research Center of Fundamental and Translational Medicine», 2 Timakova str., Novosibirsk, 630060, Russian Federation; c Novosibirsk State University, 1, Pirogova str., Novosibirsk, 630090, Russian Federation; d Lavrentyev Institute of Hydrodynamics, 15, Akademika Lavrent'yeva pr., Novosibirsk, 630090, Russian Federation; e Regional Center for Multiple Sclerosis and other autoimmune diseases of the nervous system, State Budgetary Healthcare Institution of the Novosibirsk Region "State Novosibirsk Regional Clinical Hospital" (GBUZ NSO GNOKB); 126, Nemirovich – Danchenko str., Novosibirsk, 630087, Russian Federation; f Novosibirsk State Medical University; 52, Krasny prospect av., Novosibirsk, 630091, Russian Federation

**Keywords:** CLIPPERS, Quantitative MRI, Perfusion-weighted imaging, Diffusion tensor imaging, Macromolecular proton fraction mapping, ADC, apparent diffusion coefficient, CBF, cerebral blood flow, CNS, central nervous system, CSF, cerebrospinal fluid, DOT, density of tracts, DTI, diffusion tensor imaging, DWI, diffusion-weighted imaging, FLAIR, fluid attenuated inversion recovery, ITC, International Tomography Center, MPF, macromolecular proton fraction, MS, multiple sclerosis, PWI, perfusion-weighted imaging, SWI, susceptibility-weighted imaging, WI, weighted image

## Abstract

Chronic lymphocytic inflammation with pontine perivascular enhancement responsive to steroids (CLIPPERS) is a rare chronic central-nervous-system inflammatory disorder that became known only recently, and the pathogenesis of CLIPPERS remains poorly understood. This report presents clinical and radiological features of a rare case: a young female patient who rapidly died of suspected CLIPPERS. Helpful multiparametric MRI diagnostic criteria are proposed that can help discriminate CLIPPERS from non-CLIPPERS pathologies. We reviewed clinical history, symptoms, quantitative data from brain multiparametric MRI before and after treatment, and histopathological data. Perfusion-weighted imaging revealed a decrease in regional cerebral blood flow by 31% and in cerebral blood volume by 64%, with a moderate increase in transit time and in time to peak by up to 23% in affected pontine and cerebral white matter. As estimated by diffusion tensor imaging, there was elevated density of tracts (n/mm^2^) and a decrease of fraction anisotropy (×10^−3^ mm/s^2^) in the patient's pons as compared to a healthy control: density of tracts = 13.5 vs 12.4 and fraction anisotropy = 0.32 vs 0.45, respectively. Macromolecular proton fraction values proved to be reduced (15.8% and 14.5% in the control, respectively) in the patient's cerebral peduncles by 3% and in the pons by 4.1% and in a periventricular white matter lesion by 6.4% (11.3% in the normal-looking contralateral hemisphere). Based on our findings, we argue that quantitative MRI techniques may be a valuable source of biomarkers and reliable diagnostic criteria and can shed light on the pathogenesis and exact nosological position of this disorder.

## Introduction

Chronic lymphocytic inflammation with pontine perivascular enhancement responsive to steroids (CLIPPERS) is a rare chronic central nervous system (CNS) inflammatory disorder that became known only recently (the first description of CLIPPERS dates from 2010; currently, 56 cases are reported) [1]. The pathogenesis of CLIPPERS remains poorly understood, and the nosological position of CLIPPERS has yet to be established; until now, this disorder has been described as an immune-system–mediated inflammatory process of unknown etiology [1]. CLIPPERS is a pontine-centric inflammatory disorder with distinct clinical and radiological features, such as punctate and curvilinear enhancing lesions in the pons and cerebellum [2]. Marked perivascular T-cell–mediated inflammation, a perivascular gadolinium enhancement pattern on MRI, and steroid responsiveness seem to be consistent with the (auto-) immunity-mediated nature of this condition. Nonetheless, other well-characterized diseases such as CNS lymphoma, brain stem glioma, and some autoimmune diseases may share the clinical and radiological features with CLIPPERS. The absence of formalized gold standard MRI diagnostic criteria and definitive biomarkers of CLIPPERS as well as the scarcity of histopathological data make the diagnosis challenging [3,4]. Therefore, it is necessary to study capabilities of quantitative methods in order to search for specific markers of this rare pathology.

### Objective

In this case report, clinical and radiological features of a young female patient are presented (who rapidly died of suspected CLIPPERS) to propose helpful multiparametric MRI diagnostic criteria to discriminate CLIPPERS from non-CLIPPERS pathologies. We also aimed to review the clinical history, symptoms, quantitative data from brain multiparametric MRI before and after treatment, and neuropathological-examination results.

## Case report

### Patient history

**Fig. 1:**
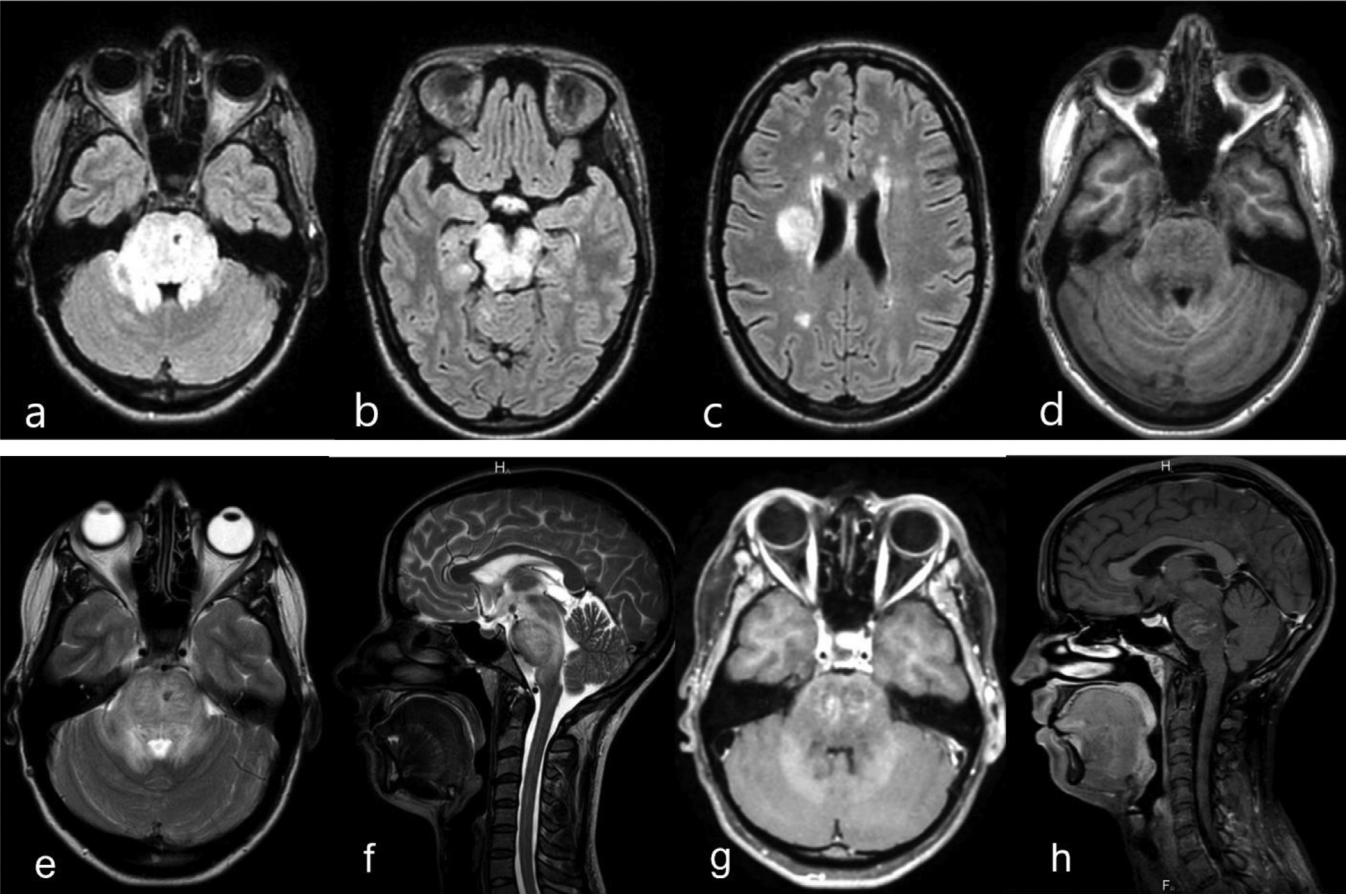
Initial representative MRI data before treatment (24 November 2021): FLAIR (a–c), T1 (d), T2 (e, f), and corresponding T1 post-gadolinium images (g, h) that resulted in a consensus diagnosis of “probable CLIPPERS.” Features of CLIPPERS pathophysiology: the T2/FLAIR high signal is much larger than enhancement, an irregular low T1 signal, associated with diffuse pontine enlargement without signs of a strong mass effect. T1 post-gadolinium data show symmetrical punctate and curvilinear enhancing lesions in the bilateral pons (arrows). No lesions or enhancing foci were noted in the cervical spine.

**Fig. 2:**
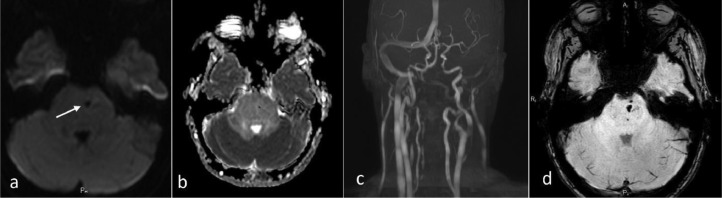
Additional representative MRI data before treatment. DWI (a), ADC-map (b), time-of-flight angiography (c) and SWI data (d) that resulted in a consensus diagnosis of “probable CLIPPERS.” Features of CLIPPERS pathophysiology: there was no restricted diffusion such as an insult or tumor, no appreciable vascular stenosis or malformations (we noted unremarkable asymmetry of vertebral arteries); deep curve vessel fragments (prominent veins) were seen on DWI and SWI (arrows).

A 33-year-old woman presented with episodes of a recurrent visual disturbance, mostly in the form of unilateral visual loss, refractory and severe headache, and aversion to some foods (namely, meat and dairy products). The patient's symptoms started in November 2021, 2 days after an unremarkable brain injury. She had no relevant medical history. Family medical history revealed that her aunt has multiple sclerosis (MS). Her initial brain MRI revealed multiple areas of curvilinear and punctate enhancement in the pons and other specific features described above, which made the diagnosis of CLIPPERS more probable (Figs. 1 and 2). After a possible infection and tumor were ruled out, she was referred for immunosuppressive therapy to a Neurological Hospital.

On 10 December 2021, the patient was hospitalized at the State Novosibirsk Regional Clinic, Neurologic department. A neurological examination showed increased tendon reflexes S>D; the finger-to-nose test and heel-to-shin tests revealed dysmetria and intention tremor, stronger on the left side. An ophthalmological examination indicated bilateral optic neuritis. Blood biochemical tests revealed elevated levels of urea and creatinine: 184.9 and 21.9 mmol/L, respectively. Consistently with persistently high blood pressure including periodic sudden spikes up to 260/180 mm Hg, a diagnosis of probable chronic glomerulonephritis was made.

Other than that, in all laboratory and serological tests, no substantial and specific aberrations were detected. A complete blood count, results of cerebral spine fluid (CSF) biochemical tests, the C-reactive protein level, and the erythrocyte sedimentation rate were unremarkable. Tests for infectious agents (appropriate serological assays in serum and CSF samples, bacterial/fungal cultures from blood and CSF, and CSF polymerase chain reaction assays) yielded no findings that would point to an infection. Immunological tests detected no autoantibodies and markers of vasculitis. Onconeuronal antibodies [Hu (ANNA 1), Yo-1 (PCA1), CV2, Ма2, Ri (ANNA2), and amphiphysin] in serum and oligoclonal bands in CSF were not detectable either.

After stabilization of blood pressure, she was prescribed dexamethasone 24, 16, and 8 mg per day for 3 days each dose. A second MRI was performed in the Neurological Hospital on 27 December 27, 2021. Unfortunately, no additional quantitative research methods were applied in the scan protocol there. Nonetheless, judging by an almost 100% reduction in T2 and a fluid attenuated inversion recovery (FLAIR) strong signal and pontine enlargement, a 3-fold reduction in the apparent diffusion coefficient in the most affected pons tissue, she experienced a really promising and rapid radiological improvement (Fig. 6).

According to almost complete radiologic resolution and clinical recovery, the patient was discharged from the hospital and was kept under observation as an outpatient starting on December 27. The patient continued to take methylprednisolone orally by herself in January 2022 at a dose of 24 mg a day. On the 21st of January, the day before a planned control multiparametric MRI, the patient suddenly died at home without any witnesses.

Neuropathological analysis revealed no remarkable macroscopic changes in the brain, except for an uneven pattern of cerebral edema. A forensic histological brain examination dated February 3, 2022, yielded some nonspecific microscopic findings such as contoured neurons, swollen basophilic nuclei, glia proliferation, dilated perivascular and pericellular spaces, and a thickening of capillary and artery walls. Even without proof from the neuropathological analysis and laboratory tests, a diagnosis of probable CLIPPERS seemed to be the most appropriate explanation during the patient's single flareup, as evidenced by the recovery caused by the clinical corticosteroid therapy, imaging characteristics on pre- and post-treatment MRI, and the subsequent death.

### Clinical imaging

The 33-year-old woman underwent brain MRI in the Medical Department of the International Tomography Center (Novosibirsk, Russia) because of visual disturbances and headache suggestive of neuritis. She had no relevant problems in her medical history. Images were acquired using a 3-T Scanner (Ingenia; Philips Medical Systems, Best, the Netherlands) with the manufacturer's transmit-receive head coil. Besides the standard structural brain MRI protocol, including T1 turbo field echo 3D and T2-weighted image (WI), we employed FLAIR 3-dimensional (3D), susceptibility-weighted imaging, diffusion-weighted imaging (DWI), 3D time-of-flight angio, post-Gad-T1 turbo field echo 3D, research sequences diffusion tensor imaging (DTI), T1 perfusion, and a fast 3D whole-brain macromolecular proton fraction (MPF) mapping protocol. The neuroimaging data were interpreted by 3 neurologists as probable CLIPPERS by consensus.

MRI of the brain uncovered disseminated and mostly symmetrical regions of distributed infiltrative aberrations localized bilaterally to the pons and to adjacent hindbrain structures (cerebellar peduncles and the mesencephalon) with similar multifocal spots in periventricular cerebral white matter manifesting T2/FLAIR signal hyperintensity (Fig. 1A-C, E, F). In the pons region, a punctate/curvilinear bilateral gadolinium enhancement on T1 (without ring enhancement) was noted: the cardinal feature of the condition in question (Fig. 1G, H). Deep curve vessel fragments (prominent veins) were seen on susceptibility-weighted imaging (Fig. 2D). A positive mass effect and secondary mild internal hydrocephaly were obvious. There was no cortical or spinal-cord involvement (Fig. 1).

There was no radiological evidence of a vascular pathology such as vasculitis or vascular lymphoma on the intra-extracranial MR-angiogram (Fig. 2C). Unremarkable vessels’ asymmetry was noted too. There was no “DWI-bright” high signal or “ADC-dark” signal corresponding to lesion-restricted diffusion (Fig. 2A, B).

**Fig. 3:**
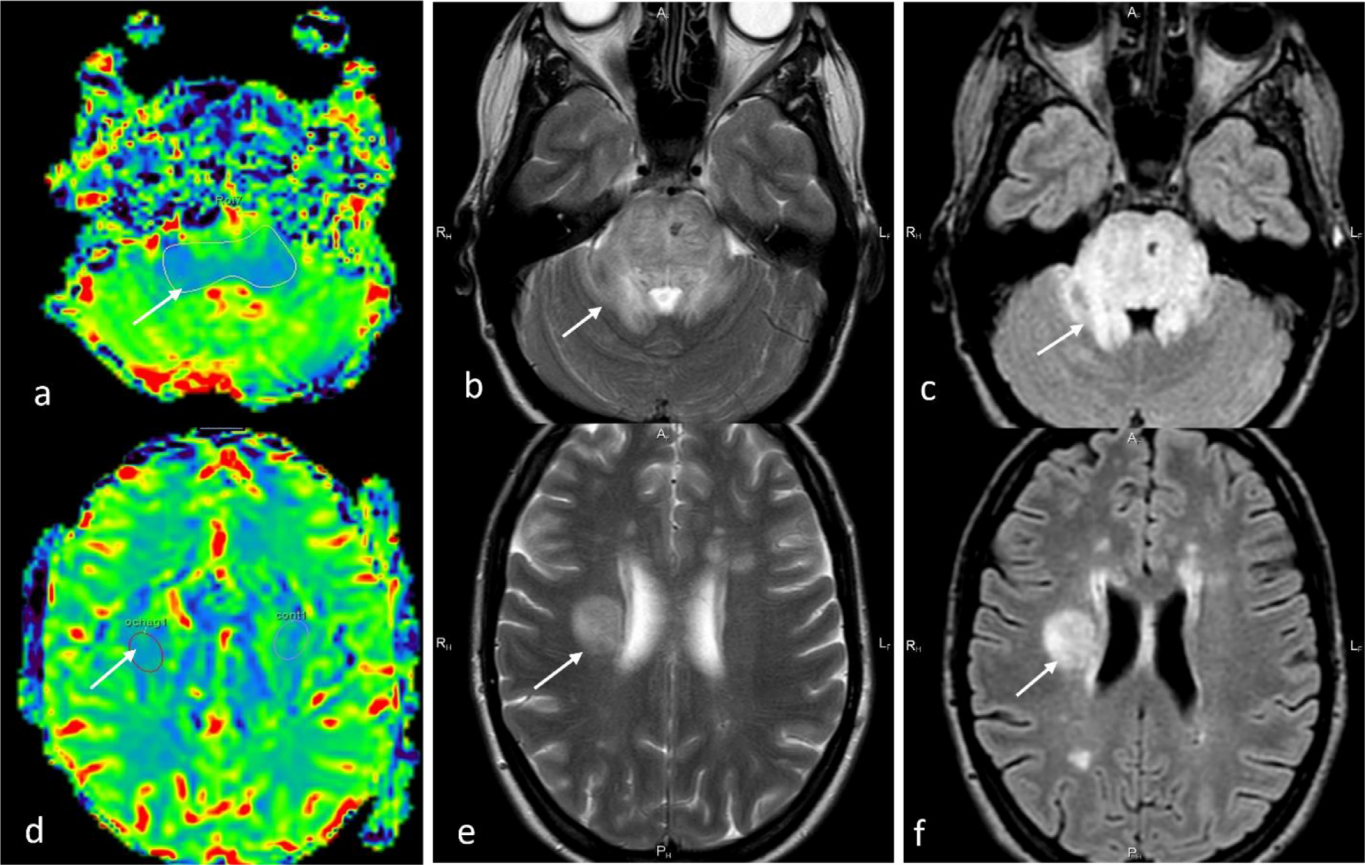
Initial PWI-derived regional CBF maps (a, d) with correlated T2 (b, e) and FLAIR (c, f) images showing decreased regional CBF in the affected pons tissue and in a white matter lesion in the right parietal lobe (CBF by 31% and cerebral blood volume by 64%).

Additional perfusion-weighted imaging (PWI) was performed in the dynamic susceptibility contrast mode: T2*-weighted 3D gradient echo planar imaging sequence (repetition time [TR]/ echo time [TE] = 1623/40 ms, matrix = 96/94, number of averages = 1, slice thickness 4 mm, number of scans = 25, acquisition time = 70 seconds), in the axial plane (contrast agent Gadovist 1 mmol/mL, 0.1 mmol/kg, 4.5 mL/s). To determine quantitative characteristics of PWI, we delineated a region of interest of round and irregular shape in the lesions, followed by automatic transfer of the geometrical template to all synchronized images. PWI-derived regional cerebral blood flow (CBF)-mapped images revealed a “blue” pattern within pons peduncles and a cerebral white matter lesion (Fig. 3A, D), contrasting with severe T2- and FLAIR “shining” (Fig. 3).

**Fig. 4:**
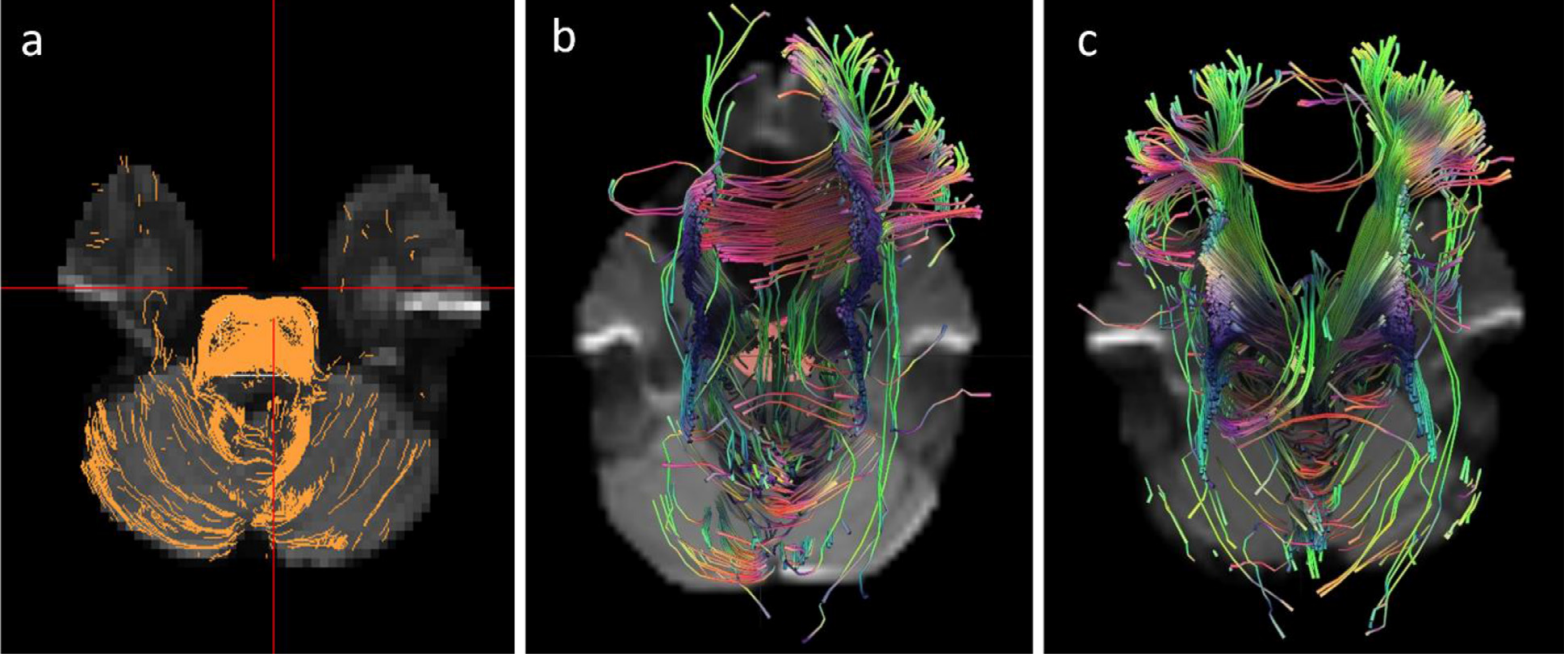
Initial DTI tractography (a). Visual representation of corticospinal tract fibers in the region of the affected hindbrain. We noticed slightly asymmetrical irregular fibers with preserved density. 3D tractography of imaged neuronal bundles as constructed through similar pons regions in the patient (b) and a healthy control subject (c).

Graphs of the “signal intensity – time” dependence in each pixel of the slice revealed a decrease in regional CBF by 31% and in cerebral blood volume by 64% with a moderate increase in transit time and time to peak up to 23% in affected pontine and cerebral white matter when compared with unaffected white matter [5]. DTI was incorporated into the scan protocol to assess the condition of neuronal fibers in the pons as the region of the most severe brain tissue anomalies. Basic DTI sequence parameters were as follows: TR/TE = 10500/73 ms, number of scans = 25, slice thickness = 2.23 mm, matrix = 96/94, field of view = 224 × 224 mm, number of directions = 32, acquisition time = 13 minutes 54 seconds. Processing and analysis of the DTI data was performed using the DSI (diffusion spectrum imaging) Studio software [6] (Fig. 4).

Visual representation of corticospinal tract fibers in the region of the affected hindbrain on initial DTI tractography showed slightly asymmetrical irregular fibers with preserved density (Fig. 4A). In the affected pons tissue, fewer neuronal bundles passing through the corona radiata and corticospinal tract were noted on the corresponding 3D tractography (Fig. 4 B, C). The density of tracts (DOT) was estimated as the ratio of the number of identified paths to the axial slice volume. The estimated DOT (n/mm^2^) and diffusion parameters (×10^−3^ mm/s^2^) in the patient's pons as compared to the healthy control were as follows: DOT = 13.5 vs 12.4, mean diffusivity = 1.05 vs 0.87, axial diffusivity fractional anisotropy = 1.42 vs 1.29, radial diffusivity = 0.87 vs 0.66, and fractional anisotropy = 0.32 vs 0.45. Considering the visual and quantitative results of DTI, the obtained data can be interpreted as an increase in interaxonal distance with intercellular edema and/or thickening of the inflamed myelin sheath.

**Fig. 5:**
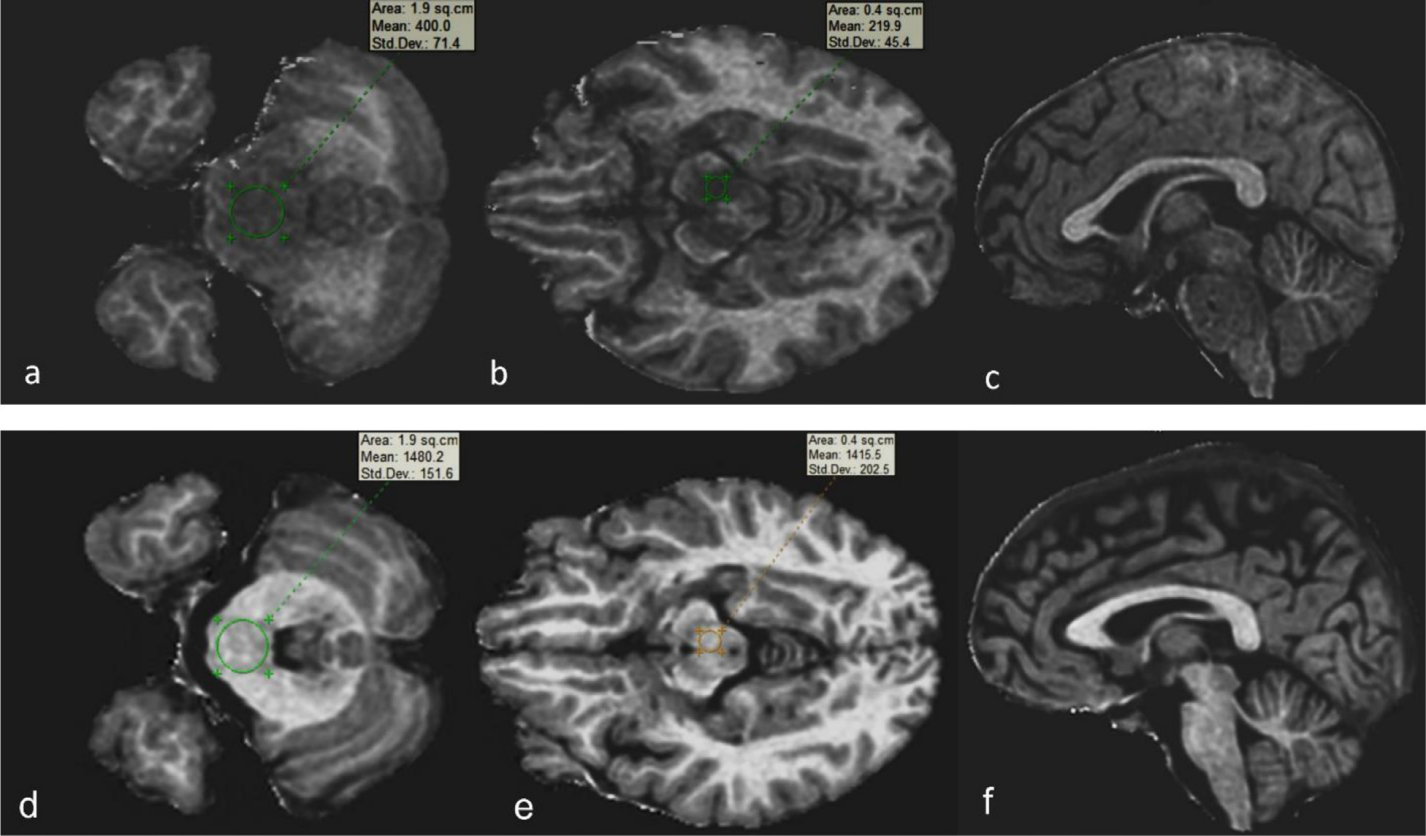
Images of an example of axial sections reconstructed from fast whole-brain 3D MPF in the patient before treatment (a–c) as compared with that of healthy women of the same age. The maps were obtained and reconstructed with the same scanner, protocol, and algorithm (d–f). A standard region of interest set (one operator) with quantitative data was utilized.

To evaluate the degree of secondary myelin loss, we carried out a new test—fast whole-brain 3D MPF mapping—well proven as a sensitive assay of myelin by several recent animal and human studies [[7], [8], [9], [10]]. We reconstructed MPF maps from magnetization transfer–weighted images and R1 maps by the single-point method [11] to compare lesions’ MPF mean ratio with normal-looking white matter in the same brain region. Because the brainstem structures were completely devoid of normal-looking tissue, we employed 3D-MPF maps of healthy women of the same age; these maps were built and reconstructed with the same scanner, protocol, and algorithm (Fig. 5).

White matter MPF was found to be reduced (from 15.8% and 14.5% in the control, respectively) in cerebral peduncles by 3% and in the pons by 4.1%; in the periventricular white matter lesion, MPF proved to be reduced by 6.4% (from 11.3% in the normal-looking contralateral hemisphere; in controls, this parameter was 18.4%) (Fig. 5).

A side benefit of the 3D-MPF mapping is the ability to perform white matter/gray matter/CSF segmentation and volume (V) calculation, which can help to evaluate lesion volume comparable data for precise assessment of dynamics of a process after treatment. According to extremely low MPF mean values in the lesion (3%- 6.4%), we propose that it belongs to the automatically generated gray matter class, and therefore the indicative lesion volume can be roughly estimated as a white matter V reduction.

In our patient, intact white matter volume was found to be reduced to 485 cm^3^ (MPF 10.2%) as compared to the control 495.8 cm^3^ (MPF 11%). Volume of gray matter with demyelinated lesions was 748 cm^3^ (MPF 5.5%) as compared to the control: 737.8 cm^3^ (MPF 5.8%). CSF volume was 251.2 cm^3^ compared to the control 330.3 cm^3^. Calculated lesion volume was ∼10.5 cm^3^ with an intact white matter MPF reduction of 7.2%.

**Fig. 6:**
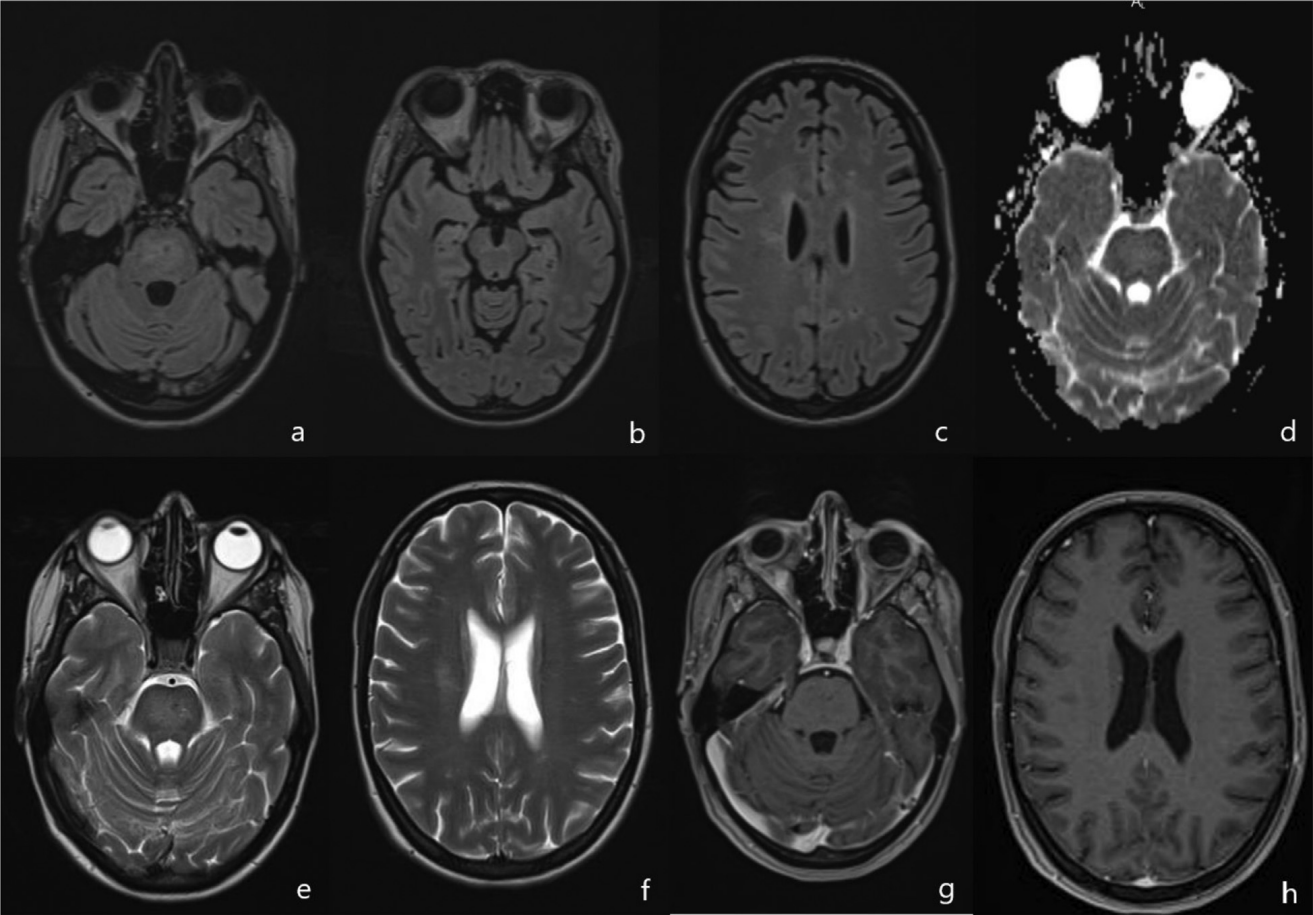
Representative MRI data after treatment with steroids (27 December 2021): FLAIR (a–c), ADC-map (d), T2 (e, f), and the corresponding T1 post gadolinium image (g, h) revealing almost complete resolution of the FLAIR signal aberration, of pontine enlargement, and of contrast enhancement after the brief course of steroids.

A second post-treatment MRI on December 27 performed at Novosibirsk State Regional Clinic showed almost complete radiologic resolution, no residual enhancement, no high signal on DWI, and no additional brain anomalies (Fig. 6).

## Discussion

In this report, we present a unique fatal case of a briefly observed clinical history of a patient as well as repeated MRI with several atypical features fulfilling revised criteria for CLIPPERS [12]. The young woman did not have typical manifestations of subacute pontocerebellar dysfunction, such as gait ataxia and diplopia. Notably, during the initial clinical manifestation, she experienced only eye-related symptoms—optic myelitis signs typical for MS—which made the diagnosis of CLIPPERS less likely. On the MRI before treatment, the lesions dominating in the infratentorial compartment, such as the cerebellum pons, were detectable with a severe mass effect that can mimic a tumor or tumor-like brain stem lesions. On the other hand, MRI enhancement characteristics on T1-WI were remarkable: punctate, bilateral, and perinodular edema without ring enhancement; these findings made a tumor less likely.

According to the distinct MRI features supported by multiparametric quantitative MRI data (PWI, DTI, and MPF mapping), well-pronounced clinical and radiological improvements induced by corticosteroid therapy, followed by rapid clinical worsening fatal to the patient, the most suitable diagnosis is CLIPPERS [13].

It has been proposed that a relapse with atypical symptoms or radiological features should be a “red flag” suggestive of a diagnosis of CLIPPERS [12]. Our patient showed an extreme and unusual discrepancy between the clinical and radiological findings, which caused us to revise the diagnosis by additional MRI quantitative techniques. The contribution of each method must be considered separately and in relation to each other to assess their significance in the differential diagnosis of CLIPPERS.

In terms of PWI, our results are consistent with another study [14], where investigators detected on PWI a global infratentorial hypoperfusion that can be characteristic of CLIPPERS. We suppose that a pronounced decrease in perfusion in the lesions can be associated with 2 possible consequences of a long-term inflammatory process: a high level of nitric oxide with secondary vasospasm and local hypoxia and a (hypometabolic) gliotic scar and areas of degenerative structural changes, which are metabolically less active and less perfused. Other researchers interpreted unexpected PWI findings of lesion hypoperfusion as dense lymphocytic inflammation with parenchymal diffuse infiltration leading to rapid atrophy, which can be considered a sign of a nonmalignant lesion. This PWI feature matched clinical findings (in a prospective study) of an extensor steroid response and instead indicated a benign process. On the other hand, we found one CLIPPERS case report showing elevated regional CBF that reached a 300% mark within a diseased pontine/curvilinear pattern of contrast enhancement [15].

According to our DTI data, the DOT in the patient's area of ​​interest (pons) was elevated to 13.5 n/mm^3^ as compared with that in a healthy volunteer: 12.5 n/mm^3^. At the same time, the indicators of average, axial, and radial diffusion also turned out to be increased, and fractional anisotropy was found to be decreased accordingly. Above the lesion under study, DTI quantitative data on specific alterations can be interpreted as a denser but less organized distribution of nerve fibers, related to an additional (inflammatory) factor in the intercellular space. Our PWI and DTI results are in agreement with known CLIPPERS biopsy features, namely predominantly T-cell infiltration in hindbrain white matter, largely perivascular in terms of distribution, and accompanied by a moderate number of histiocytes and activated microglia [11,15].

Dramatically lowered MPF mean values in lesions (2-5 times less than those in controls) indicate a remarkable myelin loss and highlight added diagnostic value of MPF mapping for ruling out subtentorial tumors containing a substantial collagen compartment because such tumors usually have high MPF values [11]. In conjunction with a sufficiently intact state and density of nerve fibers (accompanied by reversible changes in the intercellular space), the DWI, PWI, and DTI data help to rule out a malignant tumor more confidently. On the other hand, for MS with heterogeneous MPF values, fiber orientation reconstructions are most likely to be rejected based on MPF mapping and DTI data [7,16].

Simon and coworkers have also pointed out that varying degrees of myelin loss in small areas may accompany a highly intense inflammatory infiltrate (regarded as a secondary phenomenon) and focal neuronophagia, which has been observed in some cases [1,11]. Moreover, the presented case points to an excellent ability of the fast 3D-MPF mapping method to demarcate a lesion from intact white matter, thereby offering a side benefit of lesion volume calculation, which is useful for precise evaluation of dynamics of a pathological process after treatment.

Although ideally, neuropathological analysis should be performed in suspected CLIPPERS, this approach will be infeasible in many cases where vital CNS regions are affected primarily, as in our case. A blood or CSF sample of the patient during and after a flareup is not always available for antibody tests to exclude other autoimmune CLIPPERS-like diseases that should be taken into account in the differential diagnosis: autoimmune glial fibrillary acidic protein astrocytopathy [12] and neuromyelitis optica spectrum disorders [2]. Even in cases where a CSF analysis is performed in CLIPPERS, it yields inconsistent patterns, including mild pleocytosis, mildly elevated protein levels, and/or (partially transient) CSF oligoclonal bands, without malignant cells [1,17].

Accordingly, we propose to consider potential usefulness of the multiparametric and quantitative MRI radiologic features presented in our case. It was demonstrated here that these radiologic MRI features are of paramount importance for a preliminary diagnosis and for determining the severity of CLIPPERS even in the case when the only symptom (partial visual field loss) in combination with young age did not help to discriminate CLIPPERS from non-CLIPPERS, especially from MS or opticomyelitis. MRI with a whole spectrum of structural and quantitative tools remains a crucial source of diagnostic criteria for CLIPPERS that are necessary to ensure that alternative diagnoses are not overlooked and that treatment will be successful.

## Conclusion

Diagnosis of CLIPPERS is challenging and requires careful exclusion of alternative diagnoses. Typical and severe radiological features lacking clinical manifestations, marked corticosteroid responsiveness, and worsening after corticosteroid withdrawal are demonstrated in our case as hallmark features of CLIPPERS; we propose them as signs of “probable CLIPPERS.”  Because a specific serum or CSF biomarker of the disorder is currently unknown and neuropathological criteria are lacking, we suggest adding multiparametric and quantitative MRI analyses as promising techniques for intravital verification of the diagnosis; they include PWI, DTI, and MPF mapping in scan mode when CLIPPERS is suspected. We would like to emphasize that physicians should be aware of this condition and relevant differential diagnoses to take advantage of the benefits of the early diagnosis and GCS-based therapy, as in our case. Currently, extensive multimodal investigations, further studies to determine potential usefulness of quantitative MRI findings as biomarkers, and reliable diagnostic criteria are necessary; they can shed light on the pathogenesis and exact nosological position of this disorder, which remains poorly understood.

## Author contributions

All authors provided substantial contributions to the manuscript content. All authors gave final approval of the version of the article to be published.

## Ethics statement

The experiments involving human subjects were reviewed and approved by the Institutional Review Board of the International Tomography Center (the Siberian Branch of the Russian Academy of Sciences). The patient and her husband provided their written informed consent to participate in this study and for the publication of any potentially identifiable images or data included in this article.

## Patient consent

Written informed consent for publication of this case report was obtained from the patient. All images were anonymized and contain no personally identifiable information.
