# Corrigendum to “CLIPPERS: Multiparametric and quantitative MRI features” [Radiology Case Reports 18 (2023) 368–376]

**DOI:** 10.1016/j.radcr.2022.11.064

**Published:** 2022-12-25

**Authors:** Alexandra M. Korostyshevskaya, Julia A. Stankevich, Liubov M. Vasilkiv, Olga B. Bogomyakova, Denis S. Korobko, Alyona M. Gornostaeva, Andrey А. Tulupov

**Affiliations:** a The Institute International Tomography Center of the Russian Academy of Sciences, Institutskaya str., Bldg. 3а, Novosibirsk, 630090, Russian Federation; b Federal State Budgetary Scientific Institution, The Federal Research Center of Fundamental and Translational Medicine, 2 Timakova str., Novosibirsk, 630060, Russian Federation; c Novosibirsk State University, 1, Pirogova str., Novosibirsk, 630090, Russian Federation; d Lavrentyev Institute of Hydrodynamics, 15, Akademika Lavrent'yeva pr., Novosibirsk, 630090, Russian Federation; e Regional Center for Multiple Sclerosis and other autoimmune diseases of the nervous system, State Budgetary Healthcare Institution of the Novosibirsk Region "State Novosibirsk Regional Clinical Hospital" (GBUZ NSO GNOKB); 126, Nemirovich – Danchenko str., Novosibirsk, 630087, Russian Federation; f Novosibirsk State Medical University; 52, Krasny prospect av., Novosibirsk, 630091, Russian Federation

The authors would like to draw your attention to their revised Acknowledgments, which are as follows:

